# Connecting the dots: combined control of endocytic recycling and degradation

**DOI:** 10.1042/BST20180255

**Published:** 2020-12-10

**Authors:** Ewan MacDonald, Bryan Savage, Tobias Zech

**Affiliations:** 1 Institute of Systems, Molecular and Integrative Biology, University of Liverpool, Liverpool, U.K.; 2 Institut Curie, PSL Research University, Cellular and Chemical Biology unit, Endocytic Trafficking and Intracellular Delivery team, U1143 INSERM, UMR3666 CNRS, 26 rue d'Ulm, Paris Cedex 05, France.

**Keywords:** endosomal sorting, endosome, microfilaments, ubiquitin signalling

## Abstract

Endocytosis is an essential process where proteins and lipids are internalised from the plasma membrane in membrane-bound carriers, such as clathrin-coated vesicles. Once internalised into the cell these vesicles fuse with the endocytic network where their contents are sorted towards degradation in the lysosome or recycling to their origin. Initially, it was thought that cargo recycling is a passive process, but in recent years the identification and characterisation of specialised recycling complexes has established a hitherto unthought-of level of complexity that actively opposes degradation. This review will summarise recent developments regarding the composition and regulation of the recycling machineries and their relationship with the degradative pathways of the endosome.

## Introduction

**Figure 1.**
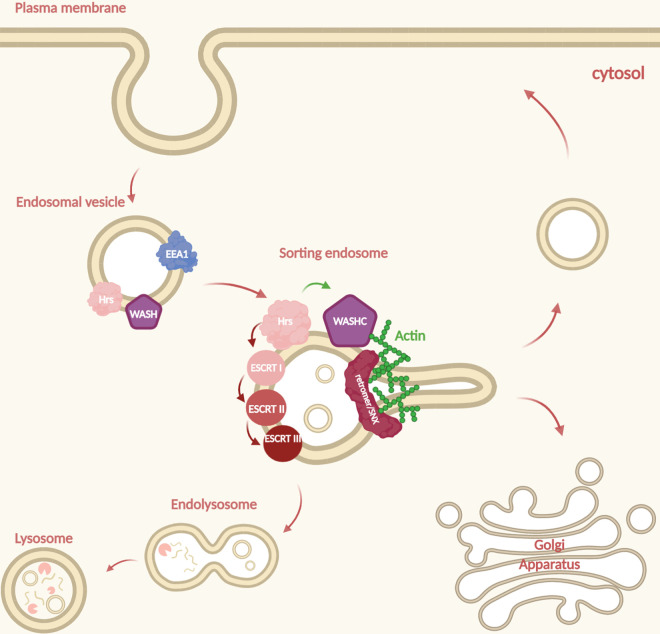
Organelles involved in endosomal trafficking. Schematic diagram of the organelles and carriers known to be involved in endocytic trafficking. Internalised cargo from the plasma membrane reaches the sorting endosome stage from which it can be transported to one of three destinations.

The endocytic network was initially identified as an important pathway for the internalisation of ligand complexes and the inducible degradation of receptors from the cell surface [1]. Subsequently, it has emerged that endocytosis is also required for the maintenance of cellular plasticity [2] and that endosomes can provide an important signalling platform, as activated receptors continue to signal and recruit downstream adapters to endosomal membranes [3]. Endocytosis occurs through a variety of clathrin dependent and independent mechanisms (discussed in detail in other reviews [4–6]), where lipids and transmembrane proteins are internalised from the plasma membrane into vesicular structures called early or sorting endosomes. There, they can either be sorted towards lysosomal degradation [7] or be recycled out to other compartments like the plasma membrane [7,8], Golgi [9,10] or specialised endosomal structures [9] (Figure 1). This balance between degradation and recycling is central to the cellular functions of the endocytic network.

Both clathrin dependent and also clathrin-independent endocytosis mechanisms such as the CLIC–GEEC (clathrin-independent carriers/GPI-anchored enriched compartments) pathway or marcopinocytosis are thought to eventually merge with early sorting endosomes [11]. The CLIC/GEEC pathway produces large tubular structures (100–150 nm) which in some cases transition through the endosomal GEEC compartment that may be a recycling component, however the mechanisms remain obscure [11]. Macropinocytosis is a specialised form of endocytosis that produces large (>0.5 µm) carriers that can adopt many of the features and machineries of the early endosome once internalised [12]. Macropinocytosis has been extensively studied in lower organisms such as Dictyostelium [13], but it also plays an important role in some mammalian cells such as macrophages [14] and many cancers up-regulate this pathway as a source of nutrient uptake by bulk internalisation of the extracellular environment [15]. This review will largely consider evidence derived from clathrin and the small clathrin-independent mechanisms of endocytosis but the reader should be aware that it may not apply fully to endosomes derived from specialised forms of endocytosis.

**Figure 2.**
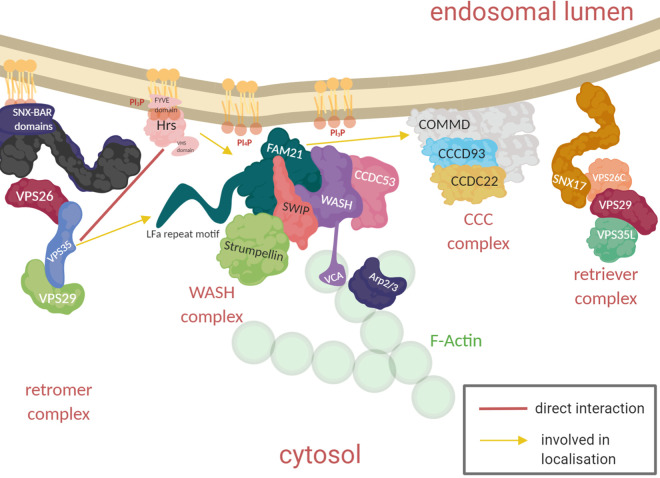
Members of the endosomal recycling machinery. Schematic representation of the protein complexes known to be involved in cargo recycling from the endosome. The spatial distribution of these complexes on the endosome is only partially known and this schematic does not reflect their sequence on endosome.

Endocytosis is a rapid process, with the entire plasma membrane of a fibroblast cell being internalised within 15 min [16]. This means that at any point, even without stimulation, a large amount of cargo enters into the endosomal system requiring correct sorting to maintain the proper homeostasis of the cell. The execution of all these events needs careful coordination of a milieu of receptors and transmembrane proteins transiting through the sorting endosome, to ensure the recycling and degradation kinetics are maintained. Historically, the protein complexes involved in degradation have been better understood than the ones involved in the recycling process [16,17]. The early consensus was that if endosomal cargo is not captured into degradative subdomains of the endosome by the ESCRT complex (endosomal sorting complex required for transport), the cargo was free to recycle through the passive flow of lipids returning to the plasma membrane. This was mainly informed by studies of the transferrin receptor, which predominantly recycles in a sequence-independent manner, in that there are no known sequences in the tail of the transferrin receptor that bind to adapters directly facilitating recycling [18]. However, several transmembrane proteins such as the beta-adrenoreceptor [19] and ci-M6PR [20] were subsequently shown to recycle in a sequence-dependent manner along specific trafficking routes, from early and late endosomes via endosomal or even trans-Golgi network (TGN) pathways [21] (Figure 1). The existence of separate trafficking routes implies that there is active sorting at the endosome in order to direct cargo to its correct destination.

The trimeric retromer (VPS26, VPS29, VPS35) was the first protein complex found to have an active recycling role [22,23]. The subsequent discovery of other retromer-like complexes [24] and the WASH complex (WASHC) [25] showed that there is potentially great complexity to receptor sorting at the endosome. The emerging picture of the endosomal sorting process is that recycling is an active process opposing degradation, by directing transmembrane proteins away from the degradation machinery. This review will outline the current understanding of how these two processes work in concert and how the balance between the two systems is altered to deal with the dynamic activation states of cargoes.

## Cargo sorting on endosomes

The distinguishing feature of the sorting endosome is its ability to direct cargo towards recycling or degradation [26]. The sorting endosome is a tubular vesicular compartment composed of a luminal body with tubules emanating from it [27,28]. It is reported to have lipid-based subdomains enriched with PI3P, that is replaced by PI3,5P and PI4P as they transition to late endosomes [29]. The architecture of the sorting endosome lends itself towards sorting with the tubular proportion concerned with the formation of vesicles, which bud away from the main endosome [30]. As the tubules have a higher ratio of membrane to volume than the lumen, it was postulated that this would favour the accumulation of receptors and effective transport [31]. This was demonstrated with mathematical modelling that predicted that the efficiency of receptor recycling was correlated with the ability of the receptor to enter into the tubule [32]. Therefore, mobility and retention of cargo into tubules can directly influence the recycling efficiency of a given transmembrane protein. These aspects can be controlled in two ways, by increasing the lifetime of the tubule or by increasing the retention of transmembrane proteins in tubules through scaffolding proteins. In an elegant study from Puthenveedu et al. [33], the authors observed that there was diversity in the life spans of tubules at the endosome and that cargoes differed in their ability to enter into tubules. They observed that TrfR was readily incorporated into tubules and therefore could recycle efficiently through short-lived tubules, while the accessibility of the beta-adrenoreceptor into these tubules was poorer, recycling predominantly through longer lived tubules that were decorated with filamentous actin (F-actin) [33]. However, the ability of tubules to stay open longer does not fully explain how cargo can be segregated into different tubules. Cargo can be clustered into recycling subdomains through sequence-dependent recycling, stretches of sequence in the tails of transmembrane proteins that bind the recycling machinery and drive recycling. Prominent examples like the ci-M6PR [20], EGFR [34], beta-adrenoreceptor [33] and the metalloproteinase MT1-MMP have all been shown to bind directly or indirectly to scaffolding proteins which facilitate their recycling by retaining transmembrane proteins into discrete subdomains.

One interesting question is that if transmembrane proteins can recycle without the aid of recycling machinery why is it necessary to have such a diversity of recycling complexes? One explanation is that incorporation into different tubules allows for a more efficient mechanism of recycling, by packing proteins with the same destination into a vesicle. Another possible function of active recycling mechanisms is that they can oppose degradation by keeping cargo away from the degradation machinery for receptors. Finally, a largely unaddressed question is the identity of vesicles that emanate from endosomes, and if different coats for the vesicles can be generated from different recycling complexes.

## Hierarchy and coupling of endosomal recycling

The key to the efficiency of sorting lies in the ability to recognise incoming cargo and specifically cluster it into distinct tubules (Figure 2). The highly conserved retromer complex trimer VPS26/29/35, and its associated sorting nexin (SNX) proteins, was the first recognised retrograde cargo trafficking complex to be discovered [22,35,36]. The retromer complex was initially shown to function in the trafficking of the acid hydrolase receptor VPS10 in yeast cells [22], and later extended to the trafficking of the mammalian homologue of VPS10, ci-M6PR and many other receptors in mammalian cells [22,23,36]. The retromer works in concert with SNXs, which which directly bind membranes through phospholipid-binding Px domains and can tubulate membranes through their BAR domain (Bin/Amphiphysin/Rvs). Together these proteins can form interactions with cargo. In yeast, there is only one SNX protein, VPS5, which forms an easily identifiable complex with the retromer trimer. This relationship was explored in detail by Lucas et al. [37] who proposed a mechanism based on the retromer trimer VPS26/29/35 with VPS5 and the transmembrane cargo DMT1 . Binding of VPS5 to VPS26 induces a conformational change in the retromer subunit VPS26 which exposes a binding site for the cargo DMT1. From a cryo-electron tomography study of yeast retromer–SNX complex on endosomal tubes, the lipid-binding SNX protein VPS5 lies close to the endosome while the retromer trimer VPS26/29/35 forms an arch bound to VPS5 [38]. The authors postulated that this ‘bent leg’ allows the retromer complex to act as a bridge coupling the lipid binding and deforming properties of the SNXs to the additional components necessary for recycling such as the WASH complex [38].

In mammalian cells the landscape is more complex, with the retromer not forming a readily identifiable complex with any SNXs. It has been proposed that the retromer trimer is associated with a changing cast of SNXs and other recycling factors [39]. SNX proteins can directly interact with cargo through bipartite motifs of ΦXΩXΦ(X)nΦ (Φ represents hydrophobic residues, X represents any residue, Ω represents aromatic residues such as Tyr or Phe and (X)n flexible n-residue linker region). For SNX5 it has been shown that it can interact with ci-M6PR and IGF-R through this mechanism and it is thought that this could be extended to many other cargoes that are known to have the necessary binding sequence [20,40,41]. The control of recycling through direct binding of SNX to cargo is conserved in yeast [40,42].

However, the additional complexity in mammalian cells has created a twist to the story, and there is now evidence that SNX5 can facilitate retrograde transport independently of the retromer through the recently proposed ESCPE-1 complex [40]. There is mounting evidence which is blurring the role of the retromer in mammalian cells, with researchers reporting no trafficking defect for ci-M6PR when genetically ablating VPS35 [20,40,41,43]. These findings directly contradict one established role for the retromer and have created controversy [44] over the exact requirements of the retromer in the control of ci-M6PR. While further experiments will be needed to bring clarity, the papers from Cullen and colleagues have highlighted two important features. The first is that there are multiple complexes, composed of a changing ensemble of proteins that are involved in recognition of specific cargoes through multiple interactions with sequences within the tail of receptors. Secondly, that a ‘one size fits all’ model when talking about different cargoes in different cell types may not be sufficient; receptors may employ multiple factors in order to recycle under different situations. In this context, the discovery of retriever [24] and the CCC complex [45] may explain some of the data surrounding the retromer. While the mechanistic workings of these complexes are currently not fully elucidated, it is clear that they are required for the recycling of many transmembrane proteins notably including the LDLR receptor [24,46]. Retriever is a novel trimeric retromer-like complex composed of DSCR3 (VPS26C), C16orf62 (VPS35L) and VPS29 working in conjunction with WASH, CCC and SNX17. It is thought to function in a similar manner to the retromer complex for cargoes such as ß1 Integrin that have been shown to recycle in a SNX dependent, but retromer independent manner [47]. The COMMD/CCDC22/CCDC93 or CCC complex is composed of CCDC22, CCDC93 and any of the other 10 COMMD proteins (COMMD1–10). Expression of COMMD1–10 proteins is variable between tissues and complexes [48] and known mutants affect recycling of the copper transporter ATP7A [45]. The CCC complex is at least partially recruited to endosomes though an interaction of its subunits CCDC22 and CCDC93 with the tail of Fam21 [49] and the retriever and CCC complexes share the common subunit VPS35L [46]. Interestingly, COMMD proteins are also implicated in NFκB signalling and its cross-talk with hypoxia-inducible factor (HIF) [50,51].

One uniting factor between the retromer and retriever complexes is their association with the endosomal localised Arp2/3 activator WASH. Loss of WASH results in loss of recycling despite intact retromer localisation [8,25] and WASH stimulates the formation of filamentous actin on the endosome by directly interacting with Arp2/3 via its VCA domain [8]. Arp2/3 facilitates the branching of existing actin filaments, creating a dynamic mesh of actin. Arp2/3 possesses little intrinsic catalytic activity and requires the action of additional actin polymerisation factors of the WASP family, such as WASH. WASH forms a pentameric complex, termed WASHC, with CCDC53, FAM21, SWIP and Strumpellin [25,52,53]. The recruitment of WASHC to the endosome is multifactorial. The ESCRT-0 component HRS is required for WASH presence on endosomes as is the tail of FAM21 that can bind the retromer complex member VPS35 [49,54]. Additional factors need to be present for the regulation of WASH including the correct lipid environment on the endosome. The Fam21 C-terminus interacts promiscuously with charged phospholipids [25,52,53] and subunits of the WASHC have been shown to interact directly with PI4KIIa [55]. Disruption of the CCC complex causes an increase in PI3P levels on the endosome because recruitment of the PI(3)P phosphatase MTMR2 is inhibited and results in increased recruitment and hyper-activation of WASHC [46]. Increases in PI4P cause the formation of actin comet tails on endosomes, presumably also because of strong activation of WASHC [56], regulated by an interaction between the ER and endosome, which also serve as sites of cleavage for tubules emanating from the endosome [57]. The tail of FAM21 is also important for the removal of the complex as perturbation of FAM21 causes an increase in the actin nucleation activity of WASH on the endosome in Dictyostelium [54,58]. Quite how FAM21 plays these dual roles is still a matter to be resolved. All this is to say that the correct endosomal environment is important for the activity of WASH. Exactly what the signals for WASHC activation are remain unknown.

How does the nucleation of filamentous actin by WASH control recycling? There are many models for WASH function. The first is that WASH polymerises an actin meshwork which can help collar receptors into recycling domains through direct and indirect interactions with actin which are present in a large number of receptors. This also has the additional function of stabilising tubules for longer leading to an increased time for receptors to accumulate within tubules [8]. The second proposal is that the action of actin can provide force on the tubules which can act to sever tubules, presumably in a friction dependent manner akin to the pinching of clathrin-independent buds at the plasma membrane [53,59]. It is entirely possible that these two models are both correct, and that WASH can have multiple functions to facilitate recycling.

## Recycling versus degradation

From early experiments observing the fate of different endocytic cargo, it was clear that internalised transmembrane proteins could arrive in the same endosomal structures, but be sorted towards different fates. The transferrin receptor (TrfR) demonstrated an ability to recycle efficiently and became a marker of the recycling pathway, while LDL and EGFR accumulated in the endosome and are ultimately sorted towards the lysosome for degradation [60–62]. Quantitative analysis of transmembrane protein internalisation and degradation demonstrated that receptor recycling occurred at a higher rate than degradation, with each LDL receptor estimated to recycle 150 times [63], TrfR 300 times [64] and asialoglycoprotein receptor 250 times [64,65]. These numbers suggest that recycling is a highly effective process that can occur with >99% efficiency. Despite this, recycling was viewed as a default and passive process. This perspective was solidified with the discovery of the ESCRT complex, a mechanistic view of transmembrane protein sorting centered around active sorting towards degradation emerged. The ESCRT complex is a large multiprotein complex which is composed of four sub complexes (ESCRT-0, ESCRT-1, ESCRT-2 and ESCRT-3) [66–68]. ESCRT functions by capturing ubiquitylated cargo at the endosome and sorting them into intra luminal vesicles (ILVs) of multivesicular bodies (MVBs) [69]. The process of internalisation of transmembrane proteins into ILVs is often considered the point of no return where captured cargo is segregated from the cytosol and is destined for degradation in the lysosome [17]. This is with the notable exception of cargo incorporated in exosomes/extracellular vesicles that are also generated from structures resembling MVBs [69,70].

The first ESCRT subcomplex to be recruited to endosomes is ESCRT-0 which is composed of HRS and STAM [71]. HRS recruitment is at least partially dependent on the interaction of PI(3)P with its VHS domain [72]. ESCRT-0 is the fulcrum for the sorting process and can modulate both the recycling and degradative pathway. The ESCRT-0 component HRS is required for the correct organisation and recruitment of the recycling machineries. On the multivesicular body, there is a clear separation of the recycling and degradative components, with each machinery occupying separate micro domains on the endosome [24,34,73]. HRS recruits a clathrin coat onto the endosomal membrane which is important for the dissociation of the ESCRT complex [74]. Alteration of the dynamics of the clathrin coat by RME-8 on the sorting endosome results in a loss of definition between different subdomains on the endosome [73].

It has been proposed by us and colleagues that, much like ubiquitin actively sorts cargo towards degradation by an affinity between ubiquitin and ubiquitin interacting motifs in the ESCRT complex, receptors can bind directly or indirectly through accessory proteins to a filamentous actin network, thus packaging cargo into recycling domains [33,34,75–77]. The affinities of receptors for both the recycling domains and ESCRT degradation domains creates a competition for sorting. This was directly tested using chimeras of the TrfR (which recycles in a sequence-independent manner) that were coupled to a non-cleavable ubiquitin and/or the actin-binding domain from MT1-MMP [78]. The addition of a cleavable ubiquitin moiety caused the TrfR to be sorted towards degradation, which was overcome by the addition of an actin-binding domain [34,79]. This shows that the addition of an actin-binding domain was able to oppose the sorting of a monoubiquitinated cargo towards degradation.

## Changes in trafficking in response to receptor activation

How the endocytic recycling system responds to dynamic changes in receptor activation is an intriguing conundrum. The response of established endosomal recycling factors has not been investigated in great detail. HRS is an interesting candidate as it has been shown to be tyrosine phosphorylated downstream of RTKs, notably the MET receptor and EGFR [80–82]. Activation of either receptor interestingly creates different phosphorylation profiles of HRS [82]. The phosphorylation of HRS controls the cytosolic to endosomal ratio of HRS, with the phosphorylated protein being dissociated from the endosome. The retention of ESCRT-0 on the membrane controls the correct formation of ILVs in a mechanism that requires the clathrin binding domain of HRS [74,83,84]. How the dynamics of activated HRS control WASH dependent recycling remains to be explored.

The activity of WASH can be directly controlled through post-translational modifications. WASHC is ubiquitylated by the MAGE-L2–TRIM27 complex that polymerises K63 linked ubiquitin chains onto K220 of the WASH subunit. K220 is found in a region that is thought to be analogous to the meander region of WAVE that stabilises the protein in an inactive state by sequestering the VCA domain though an intramolecular interaction [85,86]. Ubiquitination of K220 was shown to increase WASHC activity [85,86]. The trigger for WASHC ubiquitination in mammalian cells is currently unknown, although many signalling pathways have been identified that influence retrograde trafficking [86]. In *Drosophila* the non-receptor tyrosine kinase BTK29A phosphorylates WASH and a phospho-mimetic mutant lead to an accumulation of actin on endosomes. The equivalent mammalian kinase is yet to be identified [87]. It is also possible and probable that, like with N-WASP and WAVE, small GTPases associate with WASH and control its activity in cells. In *Drosophila* RhoA is required for WASH activation [88], however it does not appear to be required in human cells. An alternative candidate is Rac, which was shown to bind to purified WASHC *in vitro*, without affecting activation [52]. The retromer is phosphorylated and the phosphatase CDC25 is required for its function [89], though is not clear what signalling pathway could control retromer function [90]. SNX3 phosphorylation has been shown to prevent recruitment to the endosome by blocking PI3P binding [91] and phosphorylation of SNX27 downstream of cholera toxin similarly blocks its function [92].

In addition to potential changes in the recycling-degradation complex balance on endosomes, the sorting signals on the receptor themselves might change. The EGFR recycles in an actin-dependent manner, binding directly to actin through a defined ^992^YLIP motif in the cytoplasmic tail [93]. EGFR activation causes auto-phosphorylation of the tyrosine in the ^992^YLIP motif. One could postulate that phosphorylation of the receptor is necessary to prevent the receptor binding to actin and allow the receptor to engage with the ESCRT-0 complex. WASHC can direct EGFR to specialised MVBs upon stress activation by p38 MAPK, suggesting that it can be co-opted into specialised trafficking events based on receptor activation [94]. Finally, the receptor SNX27 interactions are potentially modified by receptor phosphorylation status [95].

## Perspectives-Active Recycling in the Endosomal network

- Endocytic sorting plays a critical role in cellular homeostasis and is co-opted or mutated in many disease states such as cancer metastasis or Parkinson's disease.
- Originally thought of as a passive process, recent discoveries have illuminated a diversity of protein complexes involved in endocytic recycling that are also functionally connected to factors involved in cargo degradation.
- There is much more work to be done in uncovering the mechanisms that regulate different recycling routes and how they can co-operate with activation-induced degradation of cargo.

## Abbreviations

ESCRT: endosomal sorting complex required for transport
HIF: hypoxia-inducible factor
ILVs: intra luminal vesicles
MVBs: multivesicular bodies
SNX: sorting nexin
TrfR: transferrin receptor
